# Cancer survival in Cali, Colombia: A population-based study, 1995-2004

**Published:** 2014-09-30

**Authors:** Luis Eduardo Bravo, Luz Stella García, Paola Andrea Collazos

**Affiliations:** Cali Cancer Registry. Universidad del Valle, Cali, Colombia.

**Keywords:** Cancer, cancer registries, survival analysis, stomach cancer, breast cancer, lung cancer, prostate cancer, Colombia, Latin America

## Abstract

**Background:**

There is limited information on population-based cancer survival data in Latin America.

**Objetive:**

To obtain estimates of survival for some cancers recognized as a public health priority in Colombia using data from the Cancer Registry of Cali for 1995-2004.

**Methods:**

All cancer cases for residents of Cali were included for the following sites: breast (3,984), cervix uteri (2,469), prostate (3,999), stomach (3,442) and lung (2,170). Five-year relative survival estimates were calculated using the approach described by Estève.

**Results:**

Five-year relative survival was 79% in patients with prostate cancer and 68% and 60% in women with breast or cervix uteri cancer, respectively. The cure fraction was close to zero in subjects with lung cancer and less than 10% in those with stomach cancer. The probability of dying from breast or prostate cancer in people in the lower socio-economic strata (SES) was 1.8 and 2.6 times, respectively, when compared to upper SES, *p* <0.001. Excess mortality associated with cancer was independent of age in prostate or breast cancer. After adjusting for age, sex and SES, the risk of dying from breast, cervix uteri, prostate and lung cancer during the 2000-2004 period decreased 19%, 13%, 48% and 16%, respectively, when compared with the period of 1995-1999. There was no change in the prognosis for patients with stomach cancer.

**Conclusions:**

Survival for some kinds of cancer improved through the 1995-2004 period, however health care programs for cancer patients in Cali are inequitable. People from lower SES are the most vulnerable and the least likely to survive.

## Abstract (es)

**Antecedentes:**

En Latinoamérica existe poca información de estimaciones de supervivencia al cáncer basadas en estudios de población.

**Objetivo:**

obtener estimaciones de supervivencia relativa (SR) para algunos tipos de cáncer reconocidos como prioridad de salud pública en Colombia con la información del Registro Poblacional de Cáncer de Cali.

**Métodos:**

Se incluyeron todos los casos de cáncer invasivo ocurridos en residentes de Cali durante el periodo 1994-2005 para las siguientes localizaciones: mama (3,984), cérvix (2,469), próstata (3,999 estómago (3,442) y pulmón (2,170). Las estimaciones de supervivencia relativa a 5 años se calcularon utilizando el método descrito por Estève.

**Resultados:**

La SR a cinco años fue 79% en pacientes con cáncer de próstata y 68% y 60% en mujeres con cáncer de mama y cérvix. La fracción de curación fue cercana a cero en sujetos con cáncer de pulmón y menor al 10% en aquellos con cáncer de estómago; en estos pacientes; la SR a 5 años fue <20%. La probabilidad de morir por cáncer de mama o próstata en personas de los ESE más bajos fue de 1.8 y 2.6 veces, respectivamente, en comparación con los ESE altos, *p* <0.001. Después de ajustar por edad, sexo y ESE, el riesgo de morir por cáncer de mama, cérvix, próstata o pulmón en el período 2000-2004 se redujo 19%, 13% 52% y 16%, respectivamente, en comparación con el período 1995-1999. No hubo cambio en el pronóstico para los pacientes con cáncer de estómago.

**Conclusión:**

La supervivencia para algunos tipos de cáncer ha mejorado a través de los años 1995-2004, pero los programas de atención para los pacientes con cáncer en Cali son inequitativos. Las personas de ESE bajos son más vulnerables y tienen menos probabilidad de sobrevivir al cáncer.

## Introduction

Determining the impact of cancer on the population can only be done based on the availability of information on the incidence, mortality and survival of individual patients 1. The population-based survival of individuals diagnosed with cancer is considered an important indicator of the effectiveness of the cancer care systems. Survival is the result of the access of individuals in a timely manner to the most effective treatment 2. On the other hand, the comparison of survival estimates between different malignancy locations in different regions or countries may indicate to what extent it is still possible to improve the care provided 3. 

Five-year relative survival estimates vary between 80% or higher in North America, Sweden, Japan, Finland and Australia to less than 60% in Brazil, Slovakia and below 40% in Algeria. Among the 24 European countries in the CONCORD study, the range of variation in relative survival (RS) was 70-79% 4. These significant differences in cancer survival between countries and regions 5 reflect different socio-economic factors, attitudes and practices, natural histories and efficiency of health services to provide early diagnosis, timely treatment and follow-up care 6. 

In Latin America there is limited information on population-based cancer survival 7. The majority of published studies analyze survival from different cancer locations in groups of individuals served at designated hospitals, often with the objective of comparing treatment regimens. Studies of hospital-based survival are important tools to enable a determination of the effectiveness of therapeutic regimens in the prognosis of patients at the time of diagnosis, but are characterized as presenting a selection bias, and do not fully exploit the epidemiological diversity that characterize all cases drawn from a population 8 ^,^ 9. There is thus an important gap in our understanding of the behavior of cancer survival in the population of Colombia and Latin America.

The objective of this research was to obtain estimates of survival for some cancers of major public health importance in Cali using data from the Cancer Registry of Cali Colombia (Registro Poblacional de Cancer de Cali, (RPCC)) during the period 1995-2004.

## Materials and Methods

Information on cancer incidence for selected locations (prostate, breast, stomach and lung) was obtained from RPCC databases. General mortality information was obtained at the Municipal Health Secretariat of Cali. Details regarding the history, objectives, logistics and coverage by RPCC have been previously described 10. Briefly, the RPCC is a population-based cancer registry established in 1962 that has continuous information on the incidence of all types of cancer for the population drawn from the urban area of Cali. The data for different types of cancer are actively collected from the sources of information at public and private health services, as well as through death certificates. Incidence data and quality indices for these data have been published previously in Cancer Incidence in Five Continents (CI5), Volume I-IX 11 ^-^ 19. For the last period (2003-2007), the percentage of cases morphologically verified was 84.5%, the mortality to incidence ratio (M:I) was 49.2%, and the percentage of records abstracted from a death certificate only (DCO) was 4.5%.

### Case definition

Analyses were based on registered cases of invasive cancer in Cali during the period 1995-2004 for the following cancer sites: breast and cervix uteri (women), prostate (men), stomach and lung (men and women). Individuals between 0-80 years of age at diagnosis were eligible for the study. Only the first primary invasive malignant tumor diagnosed for each individual was included in the principal survival analysis. Individuals with synchronous bilateral breast cancer were included and treated as single cases for the analysis. The following were excluded from the survival analysis: tumors identified as in situ, benign or of uncertain behavior, tumors detected during necropsy or with the only evidence found on the death certificate, and individuals who had another invasive malignancy prior to 1995. Those individuals with any subsequent malignant tumor occurring after 1995 were also excluded.

### Definition of event, beginning and ending date

Death from any cause was considered failure. Follow-up information on the 16,064 cancers patients registered on the RPCC for the period 1995-2004, with follow-up to 2006, was obtained by a variety of methods. The survival of each case was determined by the time difference (in days) between the date of incidence (index date) and date of death, date of last follow-up, or closing date of follow-up (31 December 2004). Five-year relative survival was estimated for two quinquennial periods, (i.e. 1995-1999; 2000-2004) and 10-year relative survival for the decade of 1995-2004. The patients who were alive at the end of the study or were lost to follow-up during the study were censored.

### Follow-up

To update the vital status and the date of last contact, a probabilistic record linkage was carried out between the RPCC information system and the following databases: a) General Mortality from the Municipal Health Department of Cali, 1984-2010 (350,525 records); b) hospital discharges from the principal sources of information for hospital levels II-III (321,328 records); c) System for Identification of Potential Beneficiaries of Social Programs (SISBEN, 2008) (1,289,287 records), and d) contributing regiment (health insured) of Valle de Cauca (2008) with 4,023,911 records. As an initial step we used the identity card number as a key for matching pairs to identify the records. In the absence of this identification we used the following attributes: first name, middle name, first last name, second last name, married name, date of birth, address and telephone number. FEBRL (Freely Extensible Biomedical Record Linkage) was used for data standardization (segmentation and cleaning) and probabilistic record linkage ("fuzzy" matching) of one or more files or data sources which did not share a unique record key or identifier. For record linkage we used a probabilistic approximation model based on Markov´s hidden chains and a deterministic approximation with rules defined by RPCC based on frequency tables, dictionaries and correction lists of names, dates, sites and locations.

The reliability of the process was verified through a manual review of the cancer morbidity forms, the individual records from the general mortality database of the population of Cali, and the hospital discharge database to define the coincidence, or lack of it, for the identification of each person. Finally, cases not updated were searched individually in the information system of the Solidarity Fund and Guarantee of the General Health Security System. The residual list of cases without pairings were sent to the National Identification Directory (3,794 records) and to the public telephone company in Cali (2,167 records). Telephone contact was made by a professional sociologist and member of the research group for information on 2,586 clinical histories.

### Geo-referencing the place of residence

By means of the EZU Enterprise Geobis® 9.0 program 20, residential placement for each participant was performed on a digital map of Cali. The barrio, neighborhood subdivisions, and socioeconomic strata were obtained and assigned according to the guidelines of the Municipal Planning Department of Cali. Of the 16,064 cases, 13,849 had at least one residential address and through this program 86.2% of participants were zoned.

### Statistical analysis

The dependent variable was the survival time for each individual tumor and the independent variables were: Age, Sex, SES and the Five-year grouping of periods. The estimated Overall Survival (OS) was estimated using the Kaplan-Meier method. Relative survival is the observed survival among the cancer patients divided by the expected survival in a comparable group of the general population. Expected survival was derived from complete life tables that contained the central death rates for the general population of Cali, Colombia by single year of age, sex, and single calendar year between 1995 and 2005. The Ederer II method 21 was utilized to estimate expected survival. The composition of the population structure by age, sex, and period was obtained from the official census published by the National Department of Statistics (DANE) for the years 1985-1993 and 2005. The population size for each year was estimated by geometric interpolation between the censuses that contained the analysis period. Age-standardized incidence/mortality rates (ASIR(w)/ASMR (w)) were calculated by the direct method, using the world standard population as reference. Age-specific incidence/mortality rates (ASIR/ASMR) and (ASIR(w)/ASMR(w)) are expressed per 100,000 persons/year 10. Relative survival up to 10 years after diagnosis was estimated from the individual tumor data by using the Hakulinen approach 22, embedded in the US National Cancer Institute's publicly accessible SEER*Stat^®^ software 23. Survival for each cancer site (all clinical stages combined) was described in terms of 1-year, 3-years, 5-years and 10-years of relative survival. The full-likelihood approach described by Estève *et al.* 24 *,*was used to estimate the relative rate ratio, with a 95% confidence interval, for excess mortality due to cancer by age, sex, calendar period and socio-economic strata. Statistical analysis was performed using STATA ^®^ 10.0.

The cure model was used to estimate the time at which the excess mortality due to cancer tends to zerox 25. Grouped relative survival data for cancer patients was extracted from the SEER*Stat survival session. The Cansurv software^®^ 26 fitted cure models (Weibull) to relative survival data from the RPCC.

## Results

During the period from 1995 to 2004, 33,417 new cancer cases were registered in the residents of Cali. Sixteen thousand sixty-four (16,064), or 48.1% of total cases, were selected for analysis from the following locations: breast (3,984), cervix uteri (2,469), prostate (3,999) and for men and women: stomach (3,442) and lung (2,170). The proportion of women was 54.7% and 47.2% for persons over the age of 65 (Table 1). Cancers described correspond to the codes from the International Classification of Diseases (ICD) 10^th^: C50, C53, C61, C16 and C34, respectively. For the described locations, no cases of cancer in children under 15 years of age were observed.

**Table 1.**
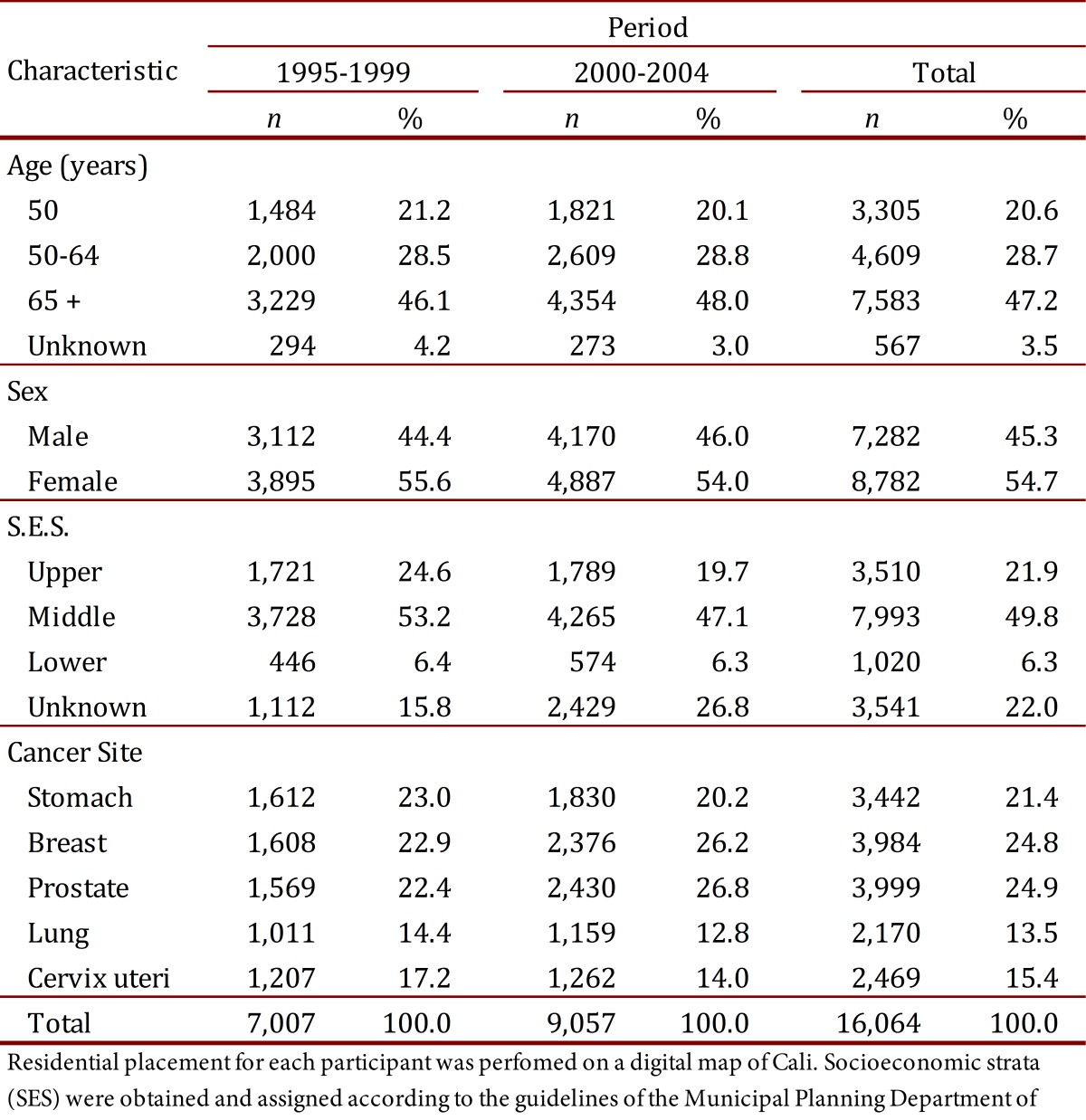
Cali, Colombia. Characteristics of patients for selected cancer sites. Comparison between two five year periods (1995-1999 and 2000-2004)**.
**

### Relative survival (RS)

Table 2 shows estimates of the RSR at one, three, five and ten years stratified by sex for two quinquennial periods (1995-1999, 2000-2004) and for the decade of 1995-2004. Cancer sites with poor 5-year relative survival (<20%) included the lung and stomach. For these tumors, relative survival was not observed to vary substantially by sex and quinquennial period.

**Table 2.**
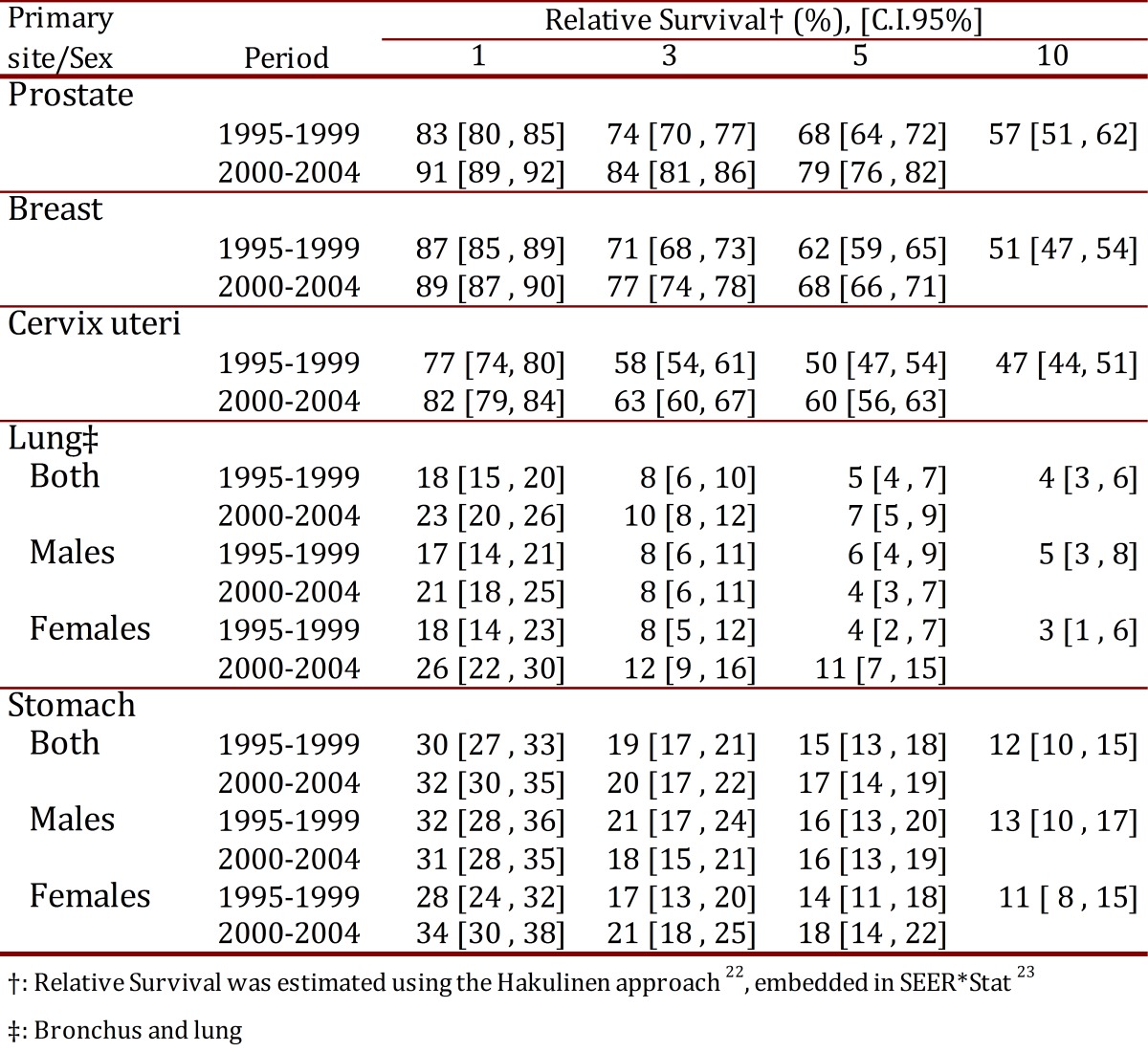
Cali, Colombia. Relative Survival Estimates† at 1, 3, 5 and 10 years for the five leading cancer sites trough 1995-2004, with follow-up to 2006.

The five-year relative survival for breast cancer and cervix uteri was 68% and 60% respectively. It was 79% for prostate cancer survival during 2000-2004. Relative survival for cervix uteri, breast cancer and prostate cancer is improving in Cali, Colombia. Five-year relative survival for breast, cervix uteri and prostate cancer increased from 62%, 50% and 68% during 1995-1999 to 68%, 60% and 79% during 2000-2004, respectively.

### Cure fractions and median survival time

For most cancers, the relative survival curve appears to plateau after several years. This plateau effect occurs when the mortality rate of the diseased individuals is the same as the expected mortality rate in the general population, or equivalently, the excess mortality rate is equal to zero, i.e. there is population cure. The cure fraction was 6% in patients with lung cancer and 15% in persons with stomach cancer. For women with breast and cervix uteri cancer the cure fraction was 45% and 58%. The median survival time (months) for uncured cases was less than one year in patients with stomach and lung cancer; and 73.2 and 18.9 in women with breast and cervix uteri cancer, respectively (Table 3).

**Table 3.**
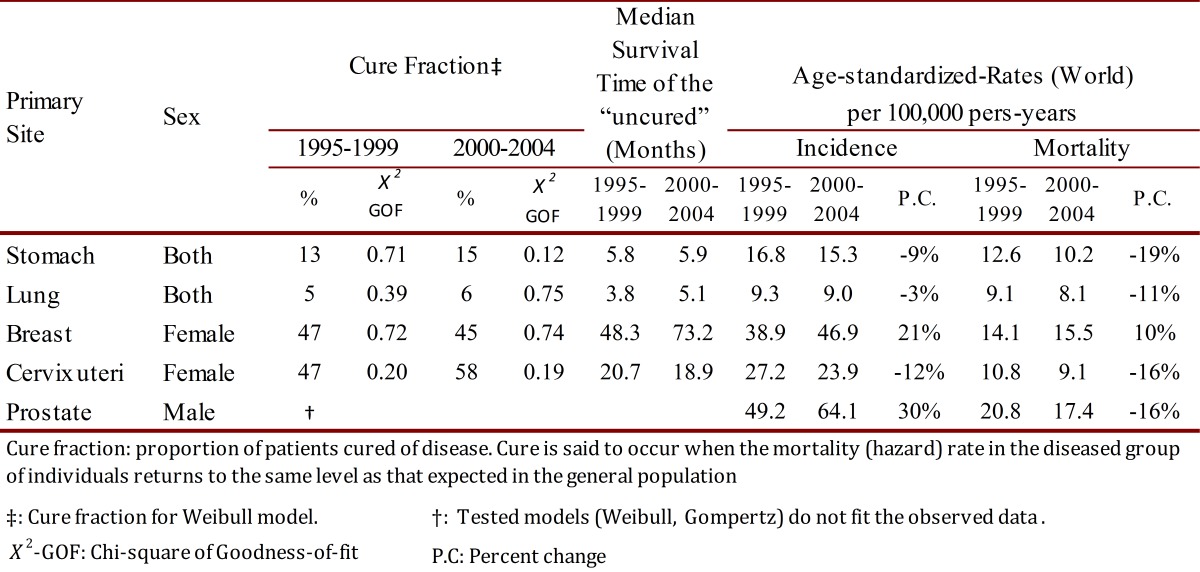
Cali, Colombia. Cure fraction (%), Median Survival Time of the "uncured", Age-standardized incidence and mortality rates for the five leading cancer sites by sex trough 1995-2004, with follow-up to 2006.

### Excess mortality due to cancer

Rate ratios for relative excess mortality by different indicators are shown in Table 4. The full-likelihood approach^24^ was used to study the relationship between survival, age, SES, and diagnostic period. For all tumors evaluated, an inverse relationship was observed between the risk of dying from cancer and the socio-economic level. The probability of dying from breast or prostate cancer in persons from lower SES was 1.8 and 2.6 times greater, respectively, when compared to upper SES, p <0.001. In the most lethal malignancies, the size of the relationship was smaller but significant. Patients from lower SES with lung or stomach cancer were at a 71% and 78% greater risk of dying when compared with patients from upper SES, p <0.001. A direct and significant relationship was observed between age and the risk of dying from lung or stomach cancer. Excess mortality associated with cancer was independent of age in prostate or breast cancer (Table 4).

**Table 4.**
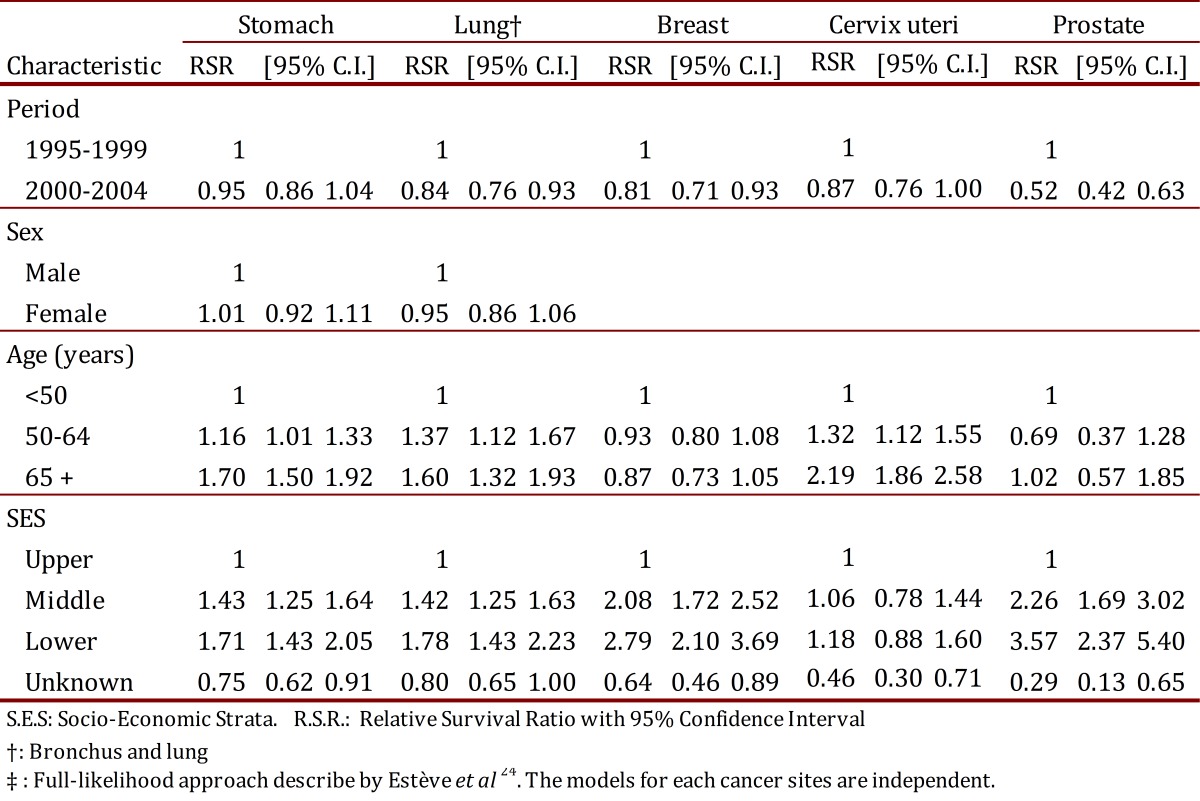
Cali, Colombia. Rate ratio, with 95% Confidence Interval, for excess mortality due to cancer by age, sex and period.

After adjusting for age, sex and socioeconomic strata, the risk of dying from breast, cervix uteri, prostate and lung cancer during the 2000-2004 period decreased 19%, 13%, 48% and 16%, respectively, when compared with the 1995-1999 period. There was no change in the prognosis for patients with stomach cancer.

## Discussion

Population estimates for survival of 16,064 people with invasive cancer in Cali, Colombia were described for the first time. This number represents 48.1% of all cases registered in Cali during the period of 1995-2004. Five-year relative survival was 79% for patients with prostate cancer, 68% and 60% for women with breast and cervix uteri cancer. Persons with lung or stomach cancer had a poor prognosis and the 5-years relative survival was less than 20%. The results of this study can be considered as representative of the average prognosis in Cali for the types of cancers studied. These findings suggest that survival for some kinds of cancer improved during the 1995-2004 period; however, programs of health care for cancer patients in Cali are inequitable and persons from lower social economic levels are the most vulnerable and the least likely to survive.

Prostate and breast cancer are a public health priority in Cali. The risk of these malignancies has increased significantly in the last 20 years 12 ^,^ 17 and, during the period evaluated, they were the leading causes of morbidity and mortality from cancer among men and women in Cali. For the period 1998-2002, the age-adjusted incidence and mortality rates per 100,000 persons per year were 47.5, and 16.2 for breast cancer and 63.2 and 18.6 for prostate cancer 12. Although the risk of these cancers is lower in comparison with Europe and the United States, survival is lower in our country, which suggests difficulties with cancer control 5.

The percent change (PC) in prostate cancer incidence and mortality rates between 2000-04 and 1995-99 was 30% and 16%, respectively. After adjusting for age, sex and socioeconomic strata, the risk of dying from prostate cancer through 2000-2004 decreased 48% when compared with the period of 1995-1999. The sudden increased incidence of prostate cancer during 1994-2004, in the absence of a similar trend in mortality rates, may reflect improvements in surveillance and early diagnostic techniques that led to diagnoses in the earlier stages where prostate cancer is more likely curable. 

Five-year relative survival estimates found in Cali are comparable to those of Costa Rica 6 Brazil and Eastern European countries and significantly lower than those described in North America, Sweden, Japan, Finland, Australia and 24 European countries in the CONCORD study 5 ^,^ 6. There are noted differences in cancer survival between countries and regions 4: U.S. survival is significantly higher than that in Europe for all solid tumors, except for those of the testes, stomach and soft tissue cancer. These differences are likely related to the intense screening orientation of the cancer control program of the United States. However, there is controversy surrounding the potential benefits from widespread usage of PSA because prostate cancer is not lethal and many so-called "early cases" would never have progressed to a fatal disease 27.

This study estimated population survival from cancers that cause the highest morbidity and mortality in Cali: prostate cancer in men and breast cancer or cervix uteri in women. For these sex-specific locations there is adequate treatment and testing available for early-stage detection; however, large differences were observed in RSR among socioeconomic strata. The probability of dying from cervix uteri, breast or prostate cancer among individuals from lower SES was 1.2, 2.8, and 3.6 times, respectively, when compared to the estimated probability for patients from upper strata, *p* <0.001. In the most lethal malignant tumors, the size of the association was smaller, but still significant. Persons from lower SES with lung or stomach cancer were at a 71% and 78% higher risk of dying compared with patients from upper SES, *p* <0.001.

Due to limitations in the availability and quality of information contained in clinical histories and/or pathology reports, it was impossible to analyze two important prognostic factors: tumor grade and stage. However, the findings described suggest that the most vulnerable persons on the social scale can suffer from inequities in the use and access to cancer services. These factors can result in delayed diagnosis and progress to more advanced stages where the odds of surviving the disease are lower when compared with higher SES patients.

In Cali, Colombia there has been a fall in the incidence of cervical cancer and a rise in incidence of breast cancer. Cervical cancer screening programs have been operational and a case-control study confirmed that screened woman have a reduced risk of disease 28. However, since overall coverage does not sufﬁciently explain all of this incidence reduction, much of it may reﬂect epidemiological transition. For other types of cancer, Colombia lacks an organized cancer screening program and early detection efforts based on opportunity and education of the population and health professionals to identify warning signs and symptoms that allow for early diagnoses 29. There is also evidence of inadequate distribution in the response to the health needs of cancer patients. The diagnosis and treatment of breast cancer is accomplished sooner in women from higher educational levels, affiliation with private health insurance plans, from the higher socioeconomic strata, and with screening. Those with private insurance plan affiliation are eight times more likely to have timely access to mammography screening than those who use the state subsidized health service 30. In lung and stomach cancer, the picture is more daunting given the lack of valid screening strategies that can be applied. 

Since 1993, Colombia has a universal health insurance program (UHIP) overseen by the Ministry of Social Protection (MSP) with a contributory and a subsidized scheme. The contributory scheme is financed by a payroll tax on formal-sector workers and a tax on employers. Unemployed, low income or informal-sector workers are financed by a government subsidy. Health policy experts have questioned the equity of this division as the contributory scheme had around double the benefits over the subsidized scheme. There is discrepancy between health insurance coverage and health care coverage and the artificial inflation of administrative and intermediation costs, absorbing 25-30% of the health system's resources. The local laws obliged the government to guarantee the right to health care for all Colombians; the private insurers shortcomings in their health-care obligations are now paid for by the government. This situation has led to sharp increases in public expenditures 31 ^,^ 32. Compared with countries of similar development, public expenditure for health in Colombia is significantly higher relative to total health spending. While more public money is channeled to the private sector, structural flaws remain untouched.

Although the study design of this investigation does not allow for the establishment of a relationship between the implementation of health insurance program and improvement in the prognoses for patients with cancer in Cali, we must point out the coincidence in timing and directionality of the changes. After adjusting for age, sex and socioeconomic status, the risk of dying from breast, prostate and lung cancer during the 2000-2004 period decreased 19%, 48% and 16%, respectively, when compared with the 1995-1999 period. No changes were observed in the survival of patients with gastric cancer and, as is in many other countries, it continues being lethal. The technological advances of modern medicine have clearly failed to impact the prognosis. Although the current health system in Colombia has contributed to greater access for the poor to health services, inequities still exist that arise from the absence of universal coverage, differences in health plans, and in the expense of the system according to population collections. The advancement of the UHIP is positive but insufficient as it has failed to achieve universal coverage and has stagnated access to services and equity.

Strengths: A population study that included the leading causes of morbidity from cancer in Cali with a sample size that corresponds to 48.1% of all cases diagnosed during the decade studied. To study the effect of the disease on survival, relative survival was estimated by taking into account the risk of mortality experienced by the population of reference. It was necessary to construct life tables for Cali from mortality database information from the Municipal Health Secretariat of Cali. The relative survival method corrects observed survival in the reference population (Cali) taking important factors, such as age, sex and period of diagnosis, into account.

Limitations include: there was no information obtained on the stage of the tumor, the RPCC was not actively tracking, and the city lacked statistics for the migrant population. In Colombia, the identification number of the citizenship card (Cédula de Ciudadanía) is not used for all medical records. Passive monitoring may be limiting, especially in less lethal malignant tumors, such as those in the breast and prostate. Life tables for Cali, according to socio-economic strata, were not available; therefore, the effect of SES on excess mortality due to cancer may be overestimated. 

Regardless, the results of this study make differences in cancer survival evident when comparison is made with developed countries. There is still much to be done to improve the care of cancer patients. It is a priority that national health authorities evaluate health promotion and prevention policies concerning oncology diseases. It is necessary to improve the coverage of programs for early detection of cancer and restructure and/or implement a network of services for the organization of support treatment and care for cancer patients, especially in the most depressed socio-economic strata.

Like other developing countries, Colombia still has to deal with the huge burden of communicable diseases, with a deficient health infrastructure and limited health budgets. In the near future it faces an escalation in the cancer burden related to population growth, population aging, and the increased risk of cancer with age. The looming cancer epidemic in our country is a topic that nobody wants to confront despite its increasing size 7.
