# Cancer survival in Cali, Colombia: A population-based study, 1995-2004

**Published:** 2015-03-30

In this article by Bravo LE and colleagues (*Colomb Med* 2014;45:110-6, 25 Jun), On page 111, under sub-section: Definition of event, beginning and ending date, the following statement is incorrect: “with follow-up to 2006,” The correct sentence should have read: “with follow-up to 2009,”.

